# Serosal complications of single-agent low-dose methotrexate used in gestational trophoblastic diseases: first reported case of methotrexate-induced peritonitis

**DOI:** 10.1038/sj.bjc.6690804

**Published:** 1999-11

**Authors:** S Sharma, S Jagdev, R E Coleman, B W Hancock, P C Lorigan

**Affiliations:** YCRC Department of Clinical Oncology, Weston Park Hospital NHS Trust, Whitham Road, Sheffield S10 2SJ, UK

**Keywords:** methotrexate, pericarditis, peritonitis, pleurisy, pneumonitis, serosal complications

## Abstract

Methotrexate (MTX) is a folate antagonist widely used both as an anticancer drug and as an immunosupressant. Administration of an 8-day methotrexate and folinic acid regime may be associated with pleuritic chest pain and pneumonitis. We have reviewed the toxicity seen in 168 consecutive patients treated with low-dose MTX for persistent trophoblastic disease. Twenty-five per cent of patients developed serosal symptoms, pleurisy was the commonest complaint. The majority of patients had mild to moderate symptoms which were controlled with simple analgesia and did not necessitate a change in treatment; 11.9% had severe symptoms which necessitated a change in treatment. One patient developed a pericardial effusion and a second patient developed severe reversible peritoneal irritation. The possible aetiology and pathophysiology of methotrexate-induced serosal toxicity is discussed. © 1999 Cancer Research Campaign


*British Journal of Cancer* (1999) **81**(6), 1037–1041
© 1999 Cancer Research Campaign
Article no. bjoc.1999.0804

### Serosal complications of single-agent low-dose methotrexate used in gestational trophoblastic diseases: first reported case of methotrexate-induced peritonitis

**S Sharma, S Jagdev, RE Coleman, BW Hancock and PC Lorigan**

YCRC Department of Clinical Oncology, Weston Park Hospital NHS Trust, Whitham Road, Sheffield S10 2SJ, UK

**Summary** Methotrexate (MTX) is a folate antagonist widely used both as an anticancer drug and as an immunosupressant. Administration of an 8-day methotrexate and folinic acid regime may be associated with pleuritic chest pain and pneumonitis. We have reviewed the toxicity seen in 168 consecutive patients treated with low-dose MTX for persistent trophoblastic disease. Twenty-five per cent of patients developed serosal symptoms, pleurisy was the commonest complaint. The majority of patients had mild to moderate symptoms which were controlled with simple analgesia and did not necessitate a change in treatment; 11.9% had severe symptoms which necessitated a change in treatment. One patient developed a pericardial effusion and a second patient developed severe reversible peritoneal irritation. The possible aetiology and pathophysiology of methotrexate-induced serosal toxicity is discussed. © 1999 Cancer Research Campaign

**Keywords**: methotrexate; pericarditis; peritonitis; pleurisy; pneumonitis; serosal complications

Methotrexate (MTX) is a folic acid analogue that inhibits DNA synthesis by causing an acute intracellular deficiency of folate coenzymes. It is used widely for the treatment of a variety of tumours and non-neoplastic conditions (Allen et al, 1986).

Methotrexate-associated pulmonary damage was first reported in 1969 and since then reports have suggested that this may occur irrespective of age, indication for use, dose or route of administration (Massin et al, 1990). However, the frequency of administration may be important as patients receiving the drug daily or weekly seem more prone to pulmonary damage than do those receiving it less frequently (Ginsberg, 1982). Features unique to the pulmonary syndrome caused by MTX are that discontinuation of the drug is not always required for clinical recovery and rechallenge with the drug may not result in recurrent symptoms. While the prognosis for pulmonary damage associated with MTX is favourable, alternative therapy may be needed because of symptoms and/or deterioration of pulmonary function (Gillespie et al, 1999).

The incidence of gestational trophoblastic disease (GTD) is 1.5 per 1000 live births. Approximately 5% of patients require treatment for persistent disease. The majority of patients have low-risk disease and can expect to be cured with chemotherapy. In the treatment of low-risk GTD, a cyclical regimen of four doses of MTX 50 mg is given intramuscularly on alternate days. Each injection is followed 24 h later by folinic acid 7.5 mg orally. Each cycle is followed by a 7-day treatment-free period. The majority of low-risk patients are cured with this regimen (Bagshawe et al, 1989).

*Received 14 April 1999*
*Accepted 30 April 1999*

*Correspondence to:* PC Lorigan

We have reviewed the serosal side-effects experienced by patients treated with single-agent low-dose intramuscular MTX for GTD between January 1988 and December 1995. We also report a probable case of MTX-induced peritonitis.

### MATERIALS AND METHODS

Between January 1988 and December 1995, 168 consecutive women with low-risk persistent GTD were treated with low-dose intramuscular MTX at this centre. Serosal toxicity of the treatment was analysed. The diagnosis of these side-effects was based on complaints of pleuritic or peritoneal pain. Pleurisy was identified as characteristic chest pain with normal chest radiograph appearance. Pneumonitis was defined as chest pain in the presence of an abnormal chest X-ray. However, these side-effects may overlap and symptoms may be associated with reduced transfer factor on pulmonary function tests in the presence of a normal chest X-ray. Differential diagnoses were excluded by clinical examinations and relevant investigations.

Symptoms were graded into two categories: mild to moderate and severe, based on the severity of the complaint. Mild to moderate symptoms were defined as those resolving spontaneously or with analgesia. Those graded as having severe toxicity were those with severe pain warranting admission to hospital if not inpatients already. In most cases of severe pain a change in treatment was required. Most patients fell into the category of mild to moderate pain.

### RESULTS

Forty-two patients (25%) treated with low-dose intramuscular MTX for persistent GTD developed serosal symptoms. Of these,

**Table 1** Serosal symptoms in 168 patients on low-dose intramuscular methotrexate between 1988 and 1995

| Symptom | Mild–moderate (%) | Severe (%) |
|---|---|---|
| Pleurisy | 34 (20.2%) | 3 (1.8%) |
| Pericarditis | 0 | 1 |
| Pneumonitis | 2 (1.2%) | 1 (0.6%) |
| Peritonitis | 0 | 1 |

pleurisy was the commonest complaint, occurring in 37 (22%) of patients treated (Table 1); 31.4% of this group were smokers and 5.7% were ex-smokers. A total of 5.7% of patients experiencing pleurisy had a history of asthma. No other significant past medical history was identified.

The vast majority of cases of MTX-induced pleurisy were of mild to moderate severity (83% of symptomatic patients). Three patients had pulmonary tests performed at the outset of treatment. Of these, one had a transfer factor of 8.5 before treatment and 7.0 afterwards (normal range 8.2–10.6), although she experienced only mild symptoms. One patient had reduced transfer factor throughout treatment (pre-treatment 6.7, post-treatment 6.2) This was associated with mild symptoms during courses one and six and severe chest pain during course five requiring admission but no change in treatment. The third had normal transfer factor throughout treatment and experienced mild chest pain during course four.

One patient complained of mild pleuritic pain while undergoing treatment but later developed pericarditis. Pneumonitis occurred in three patients, one of whom experienced severe symptoms. Pericarditis occurred in one patient and peritonitis in one. We briefly report the five patients in the severe category.

### Case report 1

A 23-year-old woman was treated with intramuscular MTX for low-risk GTD because of a persistently elevated human chorionic gondatrophin (hCG) and recurrent vaginal bleeding following two evacuations of retained products of conception (ERPC). All other staging investigations (full blood count, blood chemistry, chest X-ray, ultrasound abdomen and pelvis and computerized tomography (CT) scan of thorax) were normal as were an electrocardiogram (ECG), pulmonary function tests and transfer factor.

The temporal relationship of pleuritic chest pain to treatment was noticed from the second cycle onwards and is represented in Figure 1. She had no pain with the first cycle of treatment. The pain was not associated with shortness of breath, cough or haemoptysis. She was tachycardic but afebrile. Oxygen saturation on air was 98%. Chest and cardiovascular examination were normal. Chest X-ray, ECG and pulmonary function tests revealed no abnormality. She was converted to second-line chemotherapy and had no further symptoms.

### Case report 2

A 28-year-old woman with persistent GTD following a complete molar pregnancy was started on low-dose MTX after her serum hCG failed to show the expected fall despite three ERPCs. An

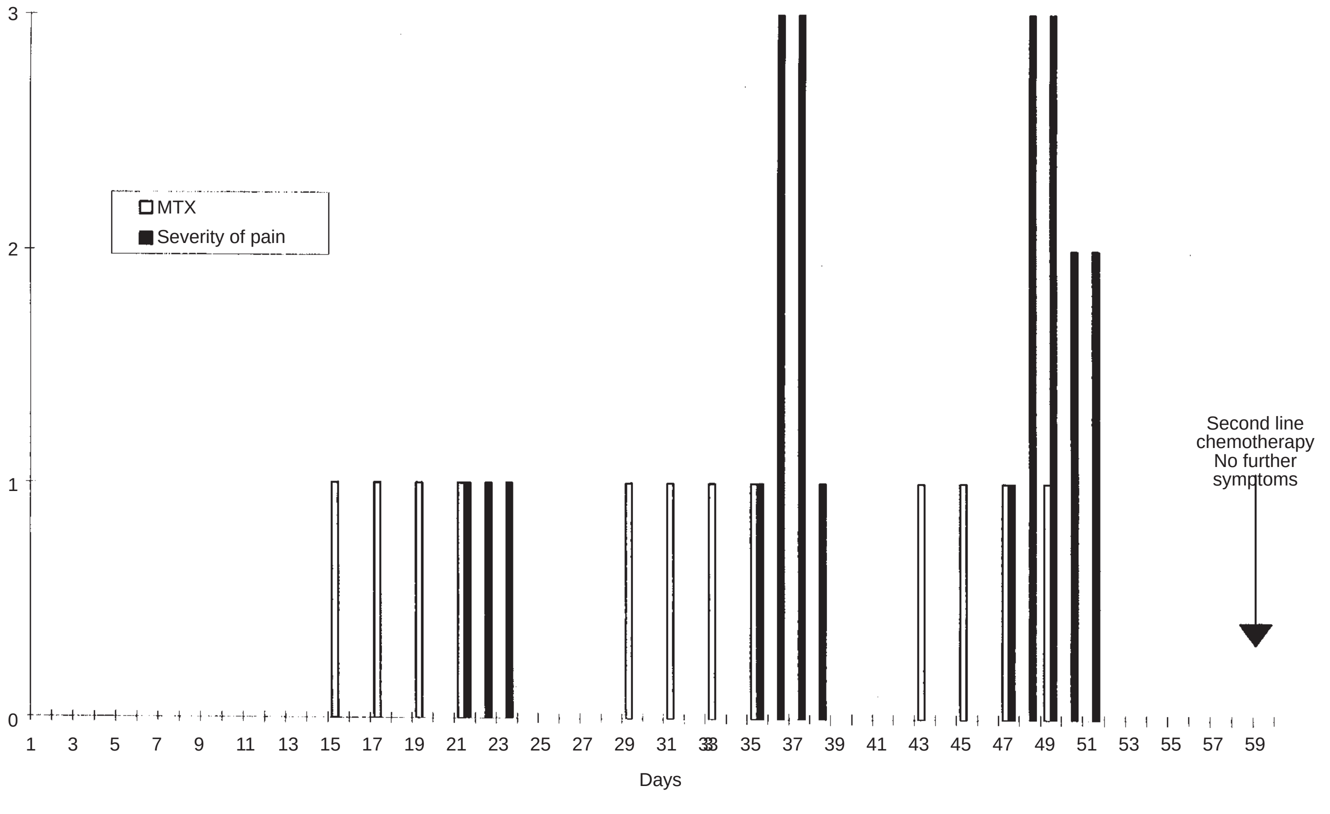

**Figure 1** Temporal pattern of pleuritis (in relation to MTX) (days 1–59). Mild, moderate and severe represented as 1, 2 and 3 units respectively.

*© 1999 Cancer Research Campaign*

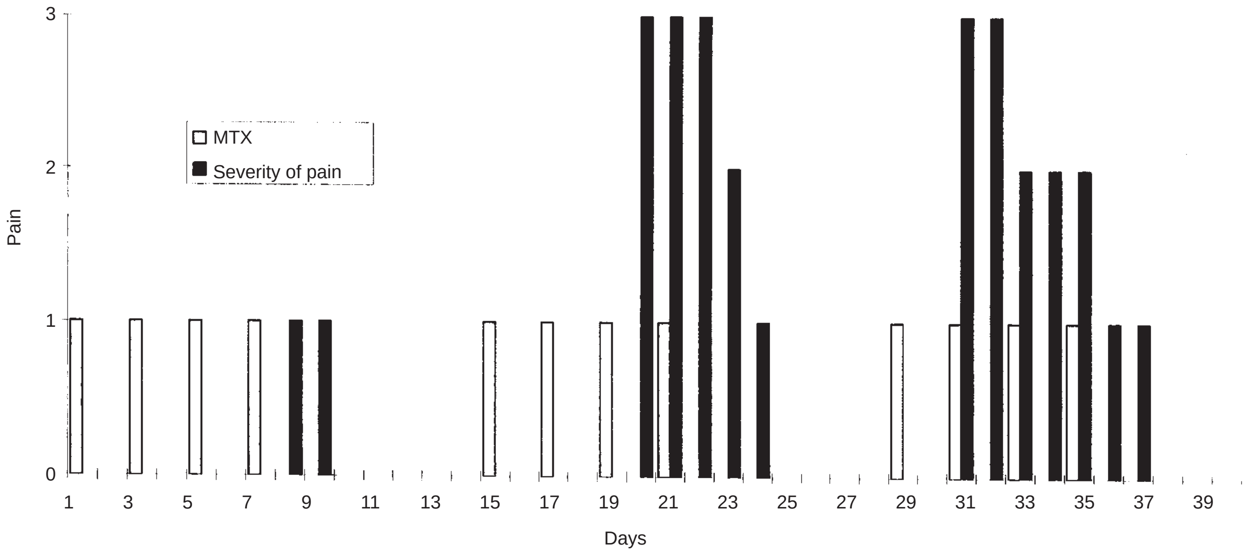

**Figure 2** Temporal pattern of pleurisy (in relation to MTX) (days 1–40). Mild, moderate and severe represented as 1, 2 and 3 units respectively.

ultrasound scan of the uterus was suggestive of persistent molar disease. Investigations revealed no evidence of lung metastases. She first complained of severe pleuritic pain in the second half of the fourth cycle. This was not associated with cough, breathlessness or haemoptysis. Clinical examination and investigations, including ventilation/perfusion scan of the lungs, were normal. The pain improved with pethidine and subsided on completion of the course. As only one further course of treatment was due, it was decided to continue with the above regimen under full analgesic cover. She tolerated the last cycle well with mild chest pain.

### Case report 3

A 25-year-old woman with persistent GTD after a complete molar pregnancy was treated with intramuscular MTX because of rising hCG. CT scan of the thorax revealed two pulmonary nodules – a 1 cm diameter nodule in left mid zone and a 5 mm nodule in right paramediastinal region. The temporal pattern of pleuritic pain during all cycles of MTX is indicated in Figure 2.

During the second cycle, she developed severe right-sided pleuritic pain not associated with cough, breathlessness or haemoptysis. Physical examination was normal. A clinical diagnosis of MTX-induced pleurisy was made. A chest X-ray was normal. She was treated with analgesia. Shortly into the third course, she was re-admitted with excruciating right-sided pleuritic chest pain. Clinical examination and chest X-ray were again normal. In view of the worsening severity of the chest pain, she was changed to second line chemotherapy of which she received seven cycles with no major untoward side-effects and no recurrence of her symptoms.

### Case report 4

A 22-year-old woman with complete molar pregnancy was treated with low-dose MTX because her hCG remained elevated 2 months after an ERPC. CT scan showed a single pulmonary nodule of 1.5 cm diameter. She tolerated the treatment with no major side-effects until the seventh cycle when she complained of mild left-sided pleuritic chest pain. The only abnormality on examination was a pleural rub at the base of the left lung. Methotrexate-associated pleurisy was diagnosed. She responded quickly to analgesia. No further investigations were done at that stage. She received a total of nine courses of methotrexate. Two months after completion of treatment, she had recurrence of her chest symptoms associated with breathlessness on exertion. Examination revealed no abnormality but chest X-ray showed cardiomegaly and echocardiogram revealed a large pericardial effusion. Her ECG was normal.

Six hundred and fifty ml of clear fluid was aspirated from her pericardial sac. Examination of the fluid was consistent with an inflammatory exudate. Bacterial cultures, serial viral titres and autoantibody screen were negative. Following pericardiocentesis, her symptoms resolved and have not recurred to date.

### Case report 5

A 27-year-old woman was admitted with persistent bleeding after a second ERPC for complete molar pregnancy. Her hCG was elevated at 62 515 iu $l^{-1}$. Ultrasound scan of the abdomen and pelvis revealed a bulky uterus consistent with persistent GTD. The right ovary measured $3.4 \times 4.5$ cm and contained cystic and solid areas. The left ovary was not visualized. CT scan of the thorax was normal.

During the first cycle of treatment, she developed right-sided abdominal pain which worsened during the next 3 days (Figure 3). The pain was aggravated by movement and was associated with tenderness and guarding. Bowel sounds were normal. Repeat ultrasound scan of abdomen and pelvis was unchanged. She was managed conservatively with appropriate analgesia.

At the start of the next cycle, she felt well in herself and her hCG had fallen to 15 839 iu $l^{-1}$. In the latter part of the second cycle, she was re-admitted with severe right-sided abdominal pain. Physical signs were similar to those found during the first cycle of treatment and further investigations including repeat ultrasound abdomen were normal. She was managed conservatively with intravenous fluid and analgesia.

*© 1999 Cancer Research Campaign*

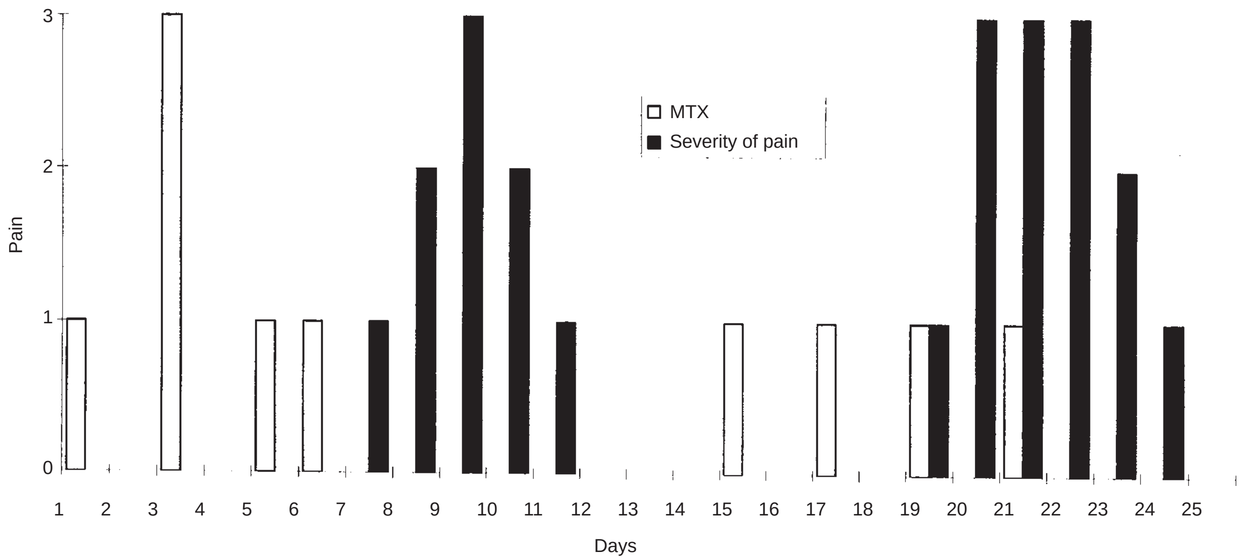

**Figure 3** Temporal pattern of peritonitis (in relation to MTX) (days 1–25). Mild, moderate and severe represented as 1, 2 and 3 units respectively.

In view of the severe abdominal pain which was related temporally to the MTX, she was changed to second-line therapy. There were no further abdominal symptoms.

### DISCUSSION

While MTX-induced pneumonitis is a well established clinical entity, the aetiology and pathophysiology are poorly understood. The syndrome of MTX pleural toxicity is usually that of pleuritic pain with both a normal chest X-ray and ventilation perfusion scan. X-ray changes, if present, are non-specific – the most common abnormality being reticulonodular shadowing, usually diffuse and symmetrical with basal predominance. Pulmonary function tests may reveal reduced gas transfer and a restrictive ventilatory defect (Forbat et al, 1995). The differential diagnosis in these patients includes pneumonia, tumour lysis in those with pulmonary metastases and pulmonary embolism. Clinical examination, chest X-ray, ECG and ventilation perfusion scan help in excluding these possible causes.

The frequency of pulmonary/pleural complications in our patients with gestational trophoblastic disease on low-dose repeated courses of methotrexate is much higher (22%) than those with other MTX regimens (5–12%) (Stover, 1992).

Patients with mild to moderate pleuritic pain are managed by maintaining adequate hydration and simple analgesia. If this does not provide adequate symptom relief stronger analgesia including opiates are used. Non steroidal anti-inflammatory drugs are avoided as they reduce renal excretion of MTX and so may exacerbate the toxicity.

The underlying pathophysiology is unclear. Akoun et al (1987) studied three patients with the syndrome and reported the synthesis of a leucocyte inhibitory factor by the peripheral blood lymphocytes after incubation with MTX in a direct leucocyte migration inhibition test. This response was not seen in the control group nor in patients on treatment who did not develop the complication, suggesting that pleurisy and pneumonitis seen with MTX is associated with a specific cellular immune response to the drug.

This hypothesis was supported by White et al (1989) who performed bronchoalveolar lavage on six patients with MTX-induced pneumonitis. They reported a significant increase in number of cells, particularly lymphocytes, in the lavage fluid. There was a predominance of helper T-cells and a relative deficiency of suppressor T-cells. These changes were not seen in the healthy control group or those on treatment with no chest symptoms. These findings suggest that MTX pneumonitis is an immunologically mediated injury rather than a direct toxic effect. Pleurisy and pneumonitis may, however, be due to different mechnisms although there may be an overlap. Mucosal toxicity has a well recognized association with MTX and yet only 4.7% of patients in this series developed mucositis. Methotrexate administration over 8 days may damage dividing mucosal or serosal cells with some selectivity for different sites.

Forbat et al (1995) suggested that a cellular immune response might cause MTX-induced pericarditis based on the findings of Case 4, although the authors indicated that the possibility of an unrecognized viral infection or a reaction to an unsuspected allergen could not be excluded.

No patients in this series developed long-term lung problems as a result of this treatment. We report a possible case of MTX-induced peritonitis. In this patient the other possible causes of abdominal pain: uterine perforation by trophoblastic disease, tumour necrosis, rupture of or bleed into an ovarian cyst, ovarian cyst torsion, pelvic inflammatory disease, acute salpingitis and other surgical/medical causes of abdominal pain were excluded as best possible. We appreciate that there exists no definitive diagnostic tool to prove our assumption and that the evidence is circumstantial. However, the temporal relationship between MTX administration and the patient's symptoms was consistent and dramatic as was the absence of symptoms in subsequent non-MTX treatments. It is therefore possible that the 'peritoneal' pain experienced by our patient was caused by a mechanism similar to that of the pneumonitis/pleurisy syndrome. Two other patients in our series had similar (though milder) symptoms: in these patients also we were unable to find another cause for the 'peritoneal' pain.

 © 1999 Cancer Research Campaign

### CONCLUSION

Serosal symptoms are a common complication of low-dose repeated MTX administration, occurring in 25% of patients treated for low-risk persistent GTD. The most common complaint is pleurisy and in the majority of cases this is of mild to moderate severity and does not require a change of treatment. Other serosal surfaces can also be involved, including pericardium and peritoneum. The underlying pathophysiology is poorly understood but severe symptoms usually respond to discontinuation of MTX and substitution of second-line therapy.

### REFERENCES

Akoun GM, Gauthier RS, Mayaud CM et al (1987) Leucocyte migration inhibition in methotrexate induced pneumonitis. Evidence of an immunological cell mediated mechanism. *Chest* **91**: 96–99

Allen J, Cooper D Jr, White DA and Matthay RA (1986) Drug induced pulmonary disease. Part I: Cytotoxic drugs. *Am Rev Resp Dis* **133**: 321–340

Bagshawe KD et al (1989) The role of low-dose methotrexate and folinic acid in gestational trophoblastic tumours (GTT). *Br J Obs Gynaecol* **96**: 795–802

Forbat LN, Hancock BW and Gershlick AH (1995) Methotrexate-induced pericarditis and pericardial efusion: first reported case. *Post Grad Med J* **71**: 244–245

Gillespie AM, Lorigan PC, Radstone CR et al (1998) Pulmonary function in patients with gestational trophoblastic disease treated with low-dose methotrexate. *Br J Cancer* **76**: 1382–1386

Ginsberg SJ and Comis RL (1982) The pulmonary toxicity of antineoplastic agents. *Semin Oncol* **9**: 34–51

Massin F, Couder B and Marot JP (1990) Methotrexate pneumonitis. *Rev Mol Resp* **7**: 5–15

Stover D (1992) Adverse effects of treatment: pulmonary toxicity. In: *Cancer: Principle and Practice of Oncology*, 4th ed, DeVita S, Hellman S and Rosenberg S (eds), pp. 2362–2365. JB Lippincott: Philadelphia

White DA, Rankin JA and Stover DL (1989) Methotrexate pneumonitis: bronchoalveolar lavage findings suggest an immunological disorder. *Am Rev Respir Dis* **139**: 18–21

*© 1999 Cancer Research Campaign*
